# Genome scan to assess the respective role of host-plant and environmental constraints on the adaptation of a widespread insect

**DOI:** 10.1186/1471-2148-9-288

**Published:** 2009-12-10

**Authors:** Stéphanie Manel, Cyrille Conord, Laurence Després

**Affiliations:** 1 Laboratoire d'Ecologie Alpine, UMR CNRS 5553, Université Joseph Fourier, 2233, rue de la Piscine, Bat D UFR de Biologie, BP 53, 38 041 Grenoble Cedex 09, France; 2 Current address: Laboratoire Population-Environnement-Développement, UMR 151 UP/IRD, Université de Provence, centre Saint-Charles, Case 10, 3, place Victor-Hugo, 13331 Marseille Cedex 3, France

## Abstract

**Background:**

The evolutionary success of phytophagous insects could result from their adaptation to different host-plants. Alternatively, the diversification of widespread species might be driven by adaptation along environmental gradients. To disentangle the respective roles of host-plant versus abiotic environmental variables acting on the genome of an oligophagous insect, we performed a genome scan using 83 unlinked AFLP markers on larvae of the large pine weevil collected on two host-plants (pine and spruce) in four forestry regions across Europe.

**Results:**

At this large geographic scale, the global genetic differentiation was low and there was no isolation by distance pattern, suggesting that migration is overwhelming genetic drift in this species. In this context, the widely used frequentist methods to detect outliers (e.g. Dfdist), which assume migration - drift equilibrium are not the most appropriate approach. The implementation of a recently developed Bayesian approach, conceived to detect outliers even in non-equilibrium situations, consistently detected 9 out of 83 loci as outliers. Eight of these were validated as outliers by multiple logistic regressions: six correlated with environmental variables, one with host-plant and one with the interaction between environmental variables and host-plant.

**Conclusion:**

These results suggest a relatively greater importance of abiotic environmental variables, as opposed to factors linked with the host-plant, in shaping genetic differentiation across the genome in this species. Logistic regression allows the nature of factors involved in locus-specific selection to be precisely identified and represents another step forward in the process of identifying adaptive loci.

## Background

Natural selection is expected to increase the frequency of locally advantageous alleles, resulting in higher among-population differentiation at these adaptive loci (measured by locus-specific *F*_ST_), compared with differentiation in the rest of the genome (neutral loci) [1,2]. Identifying adaptive divergence among populations at specific loci from genome scans is an active and challenging research area (see [3] for a review). This task is especially demanding for dominant biallelic markers such as Amplified Fragment Length Polymorphism (AFLP) markers, although they represent an easy way to scan a large number of markers scattered throughout the genome in non-model species [4-6]. So far, the most widely used method to detect outliers from AFLP genome scans is implemented in Dfdist, an extension of Fdist software that allows the use of dominant markers [7]. Dfidst is a frequentist method based on summary statistics of a symmetrical island model (i.e. drift-migration equilibrium, [8]). Each locus is compared to the neutral distribution built from the mean *F*_ST _averaged across all populations. Dfdist is likely to generate false positives (i.e. loci with higher than expected *F*_ST_) when gene flows are asymmetric across populations, and/or when some populations experiment bottlenecks. To overcome that limitation, Foll and Gaggiotti [9] have recently developed a new hierarchical Bayesian method (BayeScan) that also allows the accommodation of AFLPs data. Their method is derived from the method of Beaumont and Baldwin [10]. It produces a posterior probability for each locus being under selection. The main advantage of BayeScan is that it estimates population-specific *F*_ST _coefficients, therefore allowing for different demographic histories and different amounts of genetic drift between populations. In structured populations, the Bayesian approach is less likely to detect false positives. The proportion of false positive in detected outliers cannot be easily estimated in the Bayesian approaches as it requires simulating datasets under different scenarios [9]. By contrast, it can be estimated using false discovery rate [11] in frequentist methods.

After detecting outlier loci, it is then a challenging prospect to verify whether they are involved in local adaptation and to isolate the different ecological factors responsible for the behaviour of each outlier from a complex natural environment [4,12]. Indeed, despite recent advances in tracking adaptive genes out from the neutral background genetic variability across populations of a species, the relative role of biotic versus abiotic constraints acting on genomes in their natural environment remains a largely under-explored area. For non-model organisms, outlier loci cannot be mapped and hence their functional role will remain unknown. An alternative approach is to correlate their variation in frequency throughout the sampling area with that of continuous environmental variables, such as altitude or climate [13-15], or qualitative variables such as different host-plants for insects [6,16,17] or different life habits (limnic or benthic) for fish ecotypes [18].

The evolutionary success of phytophagous insects is thought to result mainly from their adaptation to various host-plants, with insect adaptation driving plant diversification in a co-evolutionary process [19-21]. Alternatively, the diversification of widespread species could be driven by adaptation along environmental abiotic gradients. The large pine weevil *Hylobius abietis L. *(Curculionidae) is a good model for addressing this question because it is widespread in Europe (large environmental variation) and because, during larval development, it depends exclusively on only two plant genera: spruce (*Picea*) and pine (*Pinus*). The large pine weevil is one of the most important economic pests of European conifer forests. The larvae feed under the bark of stumps and roots of recently felled trees, and take from three months to two years to develop into adults, depending on location [22], presumably because of climatic conditions and/or host plant quality [23,24]. The adults are active only under cool climatic conditions, usually in spring and autumn, and burrow into the soil during hot summers and cold winters; adults can fly large distances and can live up to four years [22]. Because of this complex life cycle, several climatic factors including temperature, precipitation, soil, frost and wind speed may have either a direct impact on larval/adult survival, or a more indirect impact on fitness through host plant quality, and represent therefore potential selective forces acting on the pine weevil genome at a large geographical scale. Adults are attracted to recently felled trees (spruce or pine) where the females lay eggs under the bark. Managed pine and spruce forests planted in Western Europe 200-300 years ago offer many oviposition opportunities for this weevil, allowing large stable populations to be sustained in contrasting climatic environments.

The first study on the population genetic structure of the pine weevils, at the European scale [25] revealed that genetic variation of this insect is better explained by geography than host-plant (5% versus 1% of total variation). Furthermore, a locus by locus AMOVA identified some loci with significant *F*_ST _across different host-plant groups, suggesting that host-plant linked selection might occur in this species. A second analysis consistently identified 2 out of 83 unlinked AFLP markers as outliers by using univariate logistic regressions to search for correlations between molecular markers scattered throughout the genome and several environmental variables suggesting an effect of climate on weevil adaptation [14]. However, the effect of the host-plant type was not tested.

**Figure 1:**
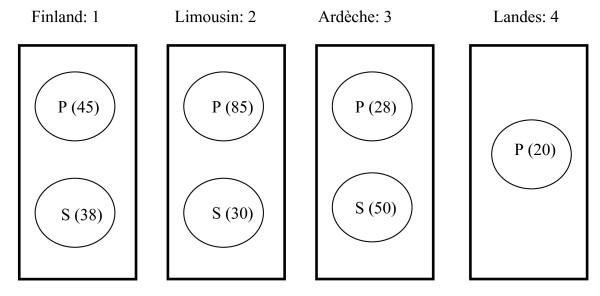
**Group-based structure of the large pine weevil larvae**. Description of the 5 group-based structures defined for the two population genetic approaches. Rectangles: regions. Circles: host-plant groups; P = Pine, S = Spruce; sample size in parentheses. Structure 1: individuals were grouped according to their geographic origin: Finland, Limousin, Ardèche, and Landes (the four rectangles 1, 2, 3, 4). Structure 2: individuals were grouped according to their geographic origin and host-plant (7 circles) Structures 3, 4, 5: within each of the three regions (rectangles 1, 2, 3) where both alternative host-plants are found (Finland, Limousin, and Ardèche), pairwise comparisons of host-plant samples were performed. Two circles in rectangle 1, 2, 3 correspond to structures 3, 4, 5 respectively.

In the present analysis, we focused on disentangling the role of abiotic environmental variables on one hand from the effect of the host-plant (pine or spruce) on the other. We therefore excluded adult weevils from the original AFLP dataset because they cannot be assigned to a host-plant, reducing the analysed dataset to 296 individual larvae. We firstly detected outlier loci across geographic and host-specific groups of individuals in 16 managed forest sites distributed across 4 large forestry regions (Table 1, Figure 1) using population genetic approaches (Dfdist and BayeScan). We then used a correlative approach to disentangle the effects of host-plants from those of various environmental variables. To further confirm the involvement of selection in genetic patterns of differentiation, we tested for the drift-migration equilibrium (i.e. isolation by distance pattern) on neutral loci. If adaptation to host-plant is promoting divergence, then we would expect to find outliers loci when comparing different host populations whereas loci candidate to diverge independently from host-plant would be rather correlated with other ecological pressures, such as climate.

**Table 1:** Geographic location, sample sizes and host-association characteristics of *Hylobius abietis *sites collected.

Site	Country-region	Longitude/latitude	Sample Size	Host-plant
1 Jalkala	Finland	27°15'E/62°33'N	10	spruce
2 Kalakukkokangas	Finland	27°15'E/62°43'N	73	mixed^1^
3 Les Quatre Vios	France - Ardèche	4°13'E/44°28'N	56	mixed^1^
4 Lachamp Raphaël	France - Ardèche	4°18'E/44°49'N	8	spruce
5 Mézilhac	France - Ardèche	4°21'E/44°48'N	10	pine
6 Etienne de Lugdares	France - Ardèche	3°57'E/44°39'N	4	spruce
7 Annouillards	France - Limousin	2°12'E/45°40'N	28	mixed^1^
8 Basville	France - Limousin	2°24'E/45°52'N	10	pine
9 Bellechassagne	France - Limousin	2°13'E/45°39'N	7	pine
10 Ebraly	France - Limousin	2°22'E/45°34'N	29	pine
11 Maussac	France - Limousin	2°09'E/45°28'N	19	pine
12 Pontgibaud	France - Limousin	2°52'E/45°49'N	10	pine
13 Puits de la Blanche	France - Limousin	2°01'E/45°17'N	7	spruce
14 Royère	France - Limousin	1°54'E/45°49'N	5	pine
15 Le Sen	France - Landes	1°30'W/44°07'N	6	pine
16 Pontenx	France - Landes	2°52'W/44°15'N	14	pine

^1 ^Both pine and spruce locally co-exist

## Results

### Population genetic approaches

The global *F*_ST_, calculated with AFLP-SURV 1.0 [26] was low but significant (*F*_ST _= 0.02, p < 0.0001). Most pairwise *F*_ST _between sites within region were non-significantly different from zero, while all pairwise *F*_ST _between the four forestry regions were significant (range 0.057-0.058, p < 0.0001), indicating that forestry region is a coherent population genetic entity. When larvae were grouped according to their forestry region (structure 1, Figure 1), four markers were consistently detected by both population genetic approaches as being under directional selection: loci 52, 38, 68 and 63 (Table 2). Dfdist detected one outlier (locus 10) that was not detected by BayeScan, and BayeScan detected two outliers (loci 13 and 47) that were not detected by Dfdist.

**Table 2:** Results of outlier detection among the 83 AFLP markers in larvae of the large pine weevil using the frequentist method Dfdist and the Bayesian inference method BayeScan.

Method of detection		Frequentist		Bayesian inference		
**Dataset**	**Locus**	***p*-value**	***F*_ST_**	**Posterior probability**	**A**	***F*_ST_**

Geography (Structure 1^1^)	**52^3^**	0.000	0.231	1	2.010	0.221
	**68**	0.000	0.338	1	1.790	0.191
	**38**	0.000	0.276	1	1.810	0.194
	10	0.000	0.248	0.758	1.000	0.105
	**63**	0.004	0.180	0.999	1.520	0.156
	13	0.099	0.061	0.974	1.320	0.136
	47	0.045	0.080	0.931	1.190	0.122

Geography + host-plant	**38**	0.000	0.259	1	2.090	0.208
(Structure 2^1^)	**52**	0.000	0.256	1	2.180	0.220
	**63**	0.000	0.225	1	1.950	0.186
	**68**	0.000	0.231	0.999	1.650	0.151
	10	0.000	0.181	0.743	0.893	0.082
	30	0.016	0.088	0.908	1.110	0.098
	33	0.018	0.095	0.876	1.030	0.091
	27	0.102	0.066	0.882	1.040	0.092
	13	0.069	0.054	0.981	1.300	0.116
	47	0.043	0.065	0.865	1.030	0.092

Local host-plant differentiation						
Regions						
Finland^2 ^(Structure 3^1^)						
						
Limousin (Structure 4^1^)	**27**	0.000	0.217	0.915	1.460	0.222
						
Ardeche^2 ^(Structure 5^1^)						

^1^As in Figure 1.

^2^No outliers were found with the significance level used.

^3^Bold type indicates markers that are detected by both methods with a type-I error (α) = 0.0006 for Dfdist, and with a posterior probability > 0.79 for BayeScan (see text for explanation about these values).

Subdividing the geographic data set into host-plant groups (pine and spruce) (structure 2, Figure 1) allowed three additional outliers, loci 27, 30 and 33, to be detected only with BayeScan (Table 2).

When local pairwise comparisons between host-plants were performed within each region (structures 3, 4, 5, Figure 1), only one locus was detected as host-specific by both genetic methods in Limousin (locus 27). This locus was also detected in host-plant groups (structure 2, Figure 1) by the Bayesian approach. No host-plant outliers were detected in Finland and in Ardèche (Table 2).

These last years, it has been suggested to use false discovery rate or *q*-values to assess significance in multiple tests [11]. The maximum estimated *q*-value among all *p*-values less than or equal to 0.0006 was 0.014: this means that among 100 loci predicted as outliers, less than 2 are false positives (i.e. neutral), so that it is very unlikely that there is any false positive among the 6 loci identified as outliers.

### Results from the correlative approach and comparison with population genetic methods

The first two axes of the principal component analysis (PCA) used to investigate correlation between environmental factors explained 96% of the variance (76% and 20% respectively), suggesting that all these variables were highly correlated (correlations of climatic variables with axis 1: 80%; correlation of altitude with axis 2: 53%). The first two axes of the PCA were used as orthogonal predictors in the logistic regressions.

Based on both a minimal Akaike criterion and the likelihood ratio test significant at level α = 6 × 10^-4^, the multiple logistic regressions identified 17 markers correlated with at least one of the 6 explanatory variable tested (Table 3). The regression coefficients of the climatic variables were significant for 14 markers, those of host-plant were significant for one marker (27), and those of interactions between host-plant and one of the PCA axis representing abiotic environmental variables for two markers (62 and 68). The maximum estimated *q*-value among all *p*-values less than or equal to 0.0006 was 0.0033. Again it is very unlikely that there is any false positive among the 17 loci identified as outliers.

**Table 3:** Results from stepwise logistic regressions relating the presence and absence of each marker to the two first axes of the principal component analysis (PCA)^1^, the host-plant (HP), and the three interactions^2^.

Markers	Axis1	Axis2	HP	Axis1 × HP	Axis2 × HP	Axis1 × Axis 2
11	**0.19** **(5.7 × 10^-4^)**	NS	NS	NS	NS	NS
13	**0.39** **(8.22 × 10^-6^)**	**-0.34** **(5.3 × 10^-4^)**	0.53 (0.15)	NS	NS	NS
19	NS	**-0.38** **(4.2 × 10^-4^)**	0.56 (0.13)	NS	NS	NS
26	0.10 (0.03)	**-0.25** **(5.4 × 10^-4^)**	0.98 (2.7 × 10^-3^)	NS	NS	NS
27	0.09 (7 × 10^-2^)	NS	**-0.96** **(1 × 10^-4^)**	**NS**	**NS**	**NS**
29	**-0.57** **(4 × 10^-4^)**	2.40 (0.24)	2.48 (0.43)	0.37 (0.45)	-2.74 (3 × 10^-3^)	NS
30	-0.19 (5.4 × 10^-3^)	**1.37** **(2.42 × 10^-5^)**	0.74 (0.59)	NS	-1.13 (1 × 10^-3^)	*NS*
33	**-0.26** **(3.64 × 10^-9^)**	NS	0.37 (0.13)	NS	NS	NS
38	**-0.45** **(4.31 × 10^-21^)**	**0.90** **(2.37 × 10^-7^)**	NS	NS	NS	NS
39	**0.16** **(3.8 × 10^-5^)**	NS	-0.090 (0.45)	0.27 (.09)	NS	NS
40	**-0.18** **(8 × 10^-6^)**	-0.26 (0.93)	0.29 (0.19)	NS	0.43 (0.10)	*NS*
42	-0.18 (0.37)	**-0.66** **(6 × 10^-5^)**	-0.74 (0.02)	0.34 (0.02)	NS	NS
52	**0.33** **(1 × 10^-7^)**	**-1.6** **(6 × 16^-16^)**	-0.22 (0.86)	NS	0.97 (1 × 10^-3^)	NS
62	-0.18 (0.37)	0.46 (4 × 10^-3^)	0.34 (0.39)	**0.31** **(3 × 10^-4^)**	NS	NS
63	**0.59** **(1 × 10^-22^)**	-0.76 (0.89)	-0.86 (0.16)	-0.18 (0.20)	0.80 (0.04)	NS

65	-0.19 (0.37)	**-0.44** **(8 × 10^-5^)**	0.45 (0.47)	0.20 (0.09)	NS	NS

68	NS	0.93 (0.05)	0.04 (0.52)	NS	**-1.05** **(4.3 × 10^-5^)**	NS

^1 ^Axis 1 and axis 2 are the two first PCA components (representing respectively 76 and 20% of total inertia) of the projection of altitude and nine climatic variables calculated from the latitude/longitude as yearly mean of monthly values for the period 1961 to 2001 (see Methods for more details).

^2^Statistically significant logistic regression coefficients are indicated in bold (*p*-values for each coefficient are given in parenthesis). NS: the correlation was not significant.

Finally, seven loci were identified as under positive selection by the regression method and at least one genetic based method (Table 2 and 3: loci 13, 27, 52, 38, 63, 30, 33 and 68). Six of these were correlated with one or two axes of the PCA representing the climatic variables, one (locus 27) was correlated with host-plant, and one (68) with the interaction between the PCA axis 2 and host-plant.

The correlative method did not detect the locus 10 detected as outlier by Dfdist but not detected by the Bayesian approach, suggesting that this is site-specific locus related to a particular demographic history of a site rather than to the ecological pressures analysed.

BayeScan suggested that loci 27, 33 and 30 were involved in host-plant adaptation, which is confirmed by logistic regression for locus 27 (Table 3). However for marker 30 and 33, the logistic regression only found an effect of environmental variables (Table 3). Finally, one locus (locus 68) detected by BayeScan both in structure 1 and 2 (Figure 1) was found significantly associated with the interaction between host-plant and PCA axis 2.

When the outliers were omitted from the dataset, there was no correlation between neutral genetic distance (*F*_ST_/(1-*F*_ST_)) and geographical distance (r = 0.35, *p *= 0.067) or the logarithm of the geographical distance (r = 0.33, *p *= 0.077).

## Discussion

### Detection of outliers - complimentary approaches

The lack of correlation between genetic and geographical distances, together with low *F*_ST _observed throughout the range of comparisons, suggest that large pine weevils populations are not at the drift-migration equilibrium; the low *F*_ST _consistently observed throughout the sampling range suggests that migration is overwhelming drift in this species. This is a further justification to use the Bayesian approach rather than Dfdist to detect outliers. Therefore we consider outliers detected by BayeScan as more reliable. However, both population genetic methods rely on the identification of loci that differ more than the rest of the genome between two or more groups of individuals. One caveat of such approaches to detect outliers is the possible detection of loci with different inherent features such as markers on sexual chromosomes, or markers that exhibit different intrinsic mutation rates. For instance, a sex-linked locus would have a different effective population size (Ne) than autosomal loci and thus more extreme *F*_ST _values; and, as a consequence, potentially mis-classified as outlier. Finally, Excoffier *et al*. [27] highlight the need to have a good understanding of the population genetic structure of the studied organism, to accurately identify loci with unusual levels of differentiation using population genetic approaches. In that context, the validation of outliers detected using population genetic approaches by correlative approaches appear to be particularly important. Eight loci were identified both by logistic regression and by BayeScan as being under positive selection. Out of these loci, one was correlated with host-plant, and one was correlated with the interaction between host-plant and abiotic factors. The congruence of the results obtained with two different methods using totally different algorithms (population genetic based vs correlative approach), makes us confident about these findings. Locus 27 was detected as an outlier linked to host-plant only in Limousin (Table 2), which raises the question of local adaptation at the regional scale. In the Limousin, locus 27 is much more frequent on spruce than on pine (0.8 versus 0.36). Although it was not detected as an outlier in the two other regions (Ardèche and Finland), it was also more frequent on spruce, suggesting that it is a global trend as also suggested by the logistic regression.

In contrast, the loci identified as outliers by only one of the methods should be interpreted with caution. First, the lack of congruence observed between the two genetic methods could be linked to the presence of false positives, which can be generated by both methods [9,10].

Second, the identification of an outlier by Dfdist but not by BayeScan could result from the fact that Dfdist does not take into account population-specific demographic effects. This was the case for one locus (locus 10) over-represented in one region (Landes) compared with the other regions (frequency 0.9 vs 0.30 to 0.39). Because the Bayesian model takes into account site-specific effects in modelling population divergence (by calculating population-specific *F*_ST_), it did not detect this locus as differing more than expected given the overall divergence of this isolated south-western region (Landes) compared to the rest of the European weevil distribution. Accordingly, the *F*_ST _estimates for this locus was strikingly different depending on the method used, with much higher estimates obtained using Dfdist than BayeScan (Table 2). Another hypothesis that can support the fact that locus 10 was only detected as an outlier by Dfdist and not BayeScan in Landes is the "allele surfing effect" [28]. In a 2D spatial demographic expansion model, it is possible that one front of the wave drift caused by a spatial bottleneck could push low frequency alleles to increase their proportion and possibly become fixed in those populations at the edge of the geographical range of species distribution. The non-identification of such locus by the logistic regression could be a way to further confirm a false positive.

### Ecological divergence: possible selective pressures

The logistic regression identified 9 loci not consistently found by the genetic methods (11, 19, 26, 29, 39, 40, 42, 62, 65). Such results could be interpreted as spurious correlations, not linked to adaptation. A second explanation could be that these loci are indeed under directional selection, but that both genetic methods failed to detect them as outliers. Indeed, both genetic methods have a high rate of false negatives, i.e. loci that are true outliers but that are not detected. For example, Beaumont & Balding [10], using simulated datasets, found that as many as 50% of true directionally selected biallelic codominant loci (selection coefficient 0.05) were mis-classified as being neutral by Fdist, a proportion that might be even higher with dominant markers. With BayeScan, Foll and Gaggiotti [9] showed that for simulated AFLP datasets with an average *F*_ST _of 0.05, 6 populations, 30 individuals sampled per population, and a selection coefficient of 0.05, the false negative rate was as high as 33%. The parameters used in these simulations were similar to those of our dataset (average *F*_ST _= 0.05, 7 populations with an average of 42 individuals sampled per population).

Two strategies can be used to search for loci under environmental selection. The first consists of applying population genetic methods to identify outliers, then using logistic regression only on the identified outliers to characterise the selective pressure that best explains their variability [17]. The second strategy is the one we used in the present study, which was also used by Joost *et al. *[14]. The approach consists of systematically applying the logistic regression to all loci to identify all marker correlations and thus perhaps identify additional markers, correlated to the environmental variable, that were not detected as outliers by the genetic approaches. As a second step in this strategy, one returns to the genetic method to investigate these newly detected relations in more depth, for example by using different groupings that better reflect the selective pressure identified by the logistic regression.

In fact, only logistic regression allows assumptions to be made on the selective pressure shaping observed patterns of adaptive genetic diversity. Although it is only a correlative approach, it can help us to formulate biological hypothesis. However, we need to keep in mind i) the possibility of spurious relations, and ii) the problem of the choice of the explanatory variables, which is not an easy task. Indeed, climatic variables are often highly inter-correlated and in most cases also correlated with elevation, longitude and latitude. In our case, to avoid the correlations between the climatic and elevation variables, we applied a PCA to produce orthogonal predictors, reducing a total of 10 highly correlated explanatory variables to two 'environmental' predictors. However, to have more precise information about the involvement of each climatic/elevation variable on the behaviour of each locus, it would be necessary to use the climatic variables directly rather than the PCA axes as explanatory factors, as done in Joost *et al*. [14]. In this later paper based on a larger dataset but not testing for the impact of the host-plant on locus-specific differentiation, two outliers (loci 38 and 52, also detected in the present analysis) were shown to be correlated with climatic variables, locus 38 being positively correlated with the number of days of ground frost and negatively with precipitation, sunshine and diurnal temperature range, while locus 52 was positively correlated with diurnal temperature range. *A priori *biological assumptions and knowledge about the selective pressure at play in nature can help with the choice of predictors to be used in the model. However, when no *a priori *assumption is available, all variables (after checking for correlations) should be included in the model. We do nevertheless need to keep in mind that it is always better to favour direct predictors (e.g. gradient of humidity) over indirect predictors (e.g. elevation) [29].

Although migration is likely to be important in this flying weevil, which may also be enhanced due to the high connectivity of pine and spruce planted forests in Europe and perhaps by human-mediated translocations, we detected 8 loci out of 83 (9.6%) under directional selection across Europe - corresponding to a selection coefficient 5 times stronger than migration [10]. Out of these adaptive loci, only one was correlated with the host-plant, and one was correlated with the interaction between host-plant and abiotic variables. *Hylobius *larvae feed on decaying plant material, so that the food quality might depend both on the host-plant type and on climatic variables. Furthermore, host-plant genotypes might vary from a region to another, explaining why the interaction between both biotic and abiotic factors might be more important than each factor taken separately in explaining genetic variability at some particular locus. This shows that in this widespread oligophagous weevil, climatic variables have a relatively larger effect than the host-plant in shaping genetic differentiation across the genome. Managed conifer forests in Western Europe are relatively recent from an evolutionary perspective and have been planted with both spruce and pine throughout large areas outside the natural range of the two tree genera, thereby possibly limiting the opportunities for selection based on host-plant. In contrast, the wide geographic range of these managed forests means that *Hylobius abietis *encounters a large variability in climatic conditions. For instance, this weevil lives underground during its diapause stage, and the length of the soil frost period is likely to be a selective factor [14]. We found only limited evidence for host-plant-specific outliers, which represented only 1.2% of all loci. This is comparable to the 1-2% of AFLP loci detected as host-specific in a genome scan performed on different ecotypes of *Timema *walking sticks, using two host-plant species [17], and slightly less than that observed across maple and willow beetles (5% of host-specific loci, [16]). Most of the host groups in this latter study were however taken from different geographic localities, making it difficult to distinguish environmental effects from host-specific adaptations. Finally, the limited evidence for host-plant adaptive loci in *Hylobius abietis *could be related to the presence of obligate endosymbiotic bacteria in the midgut of larvae [30]. Although the precise role of these bacteria in *Hylobius abietis *fitness has not yet been elucidated, the presence of this third genome (not included in our genome scan because insect gut was removed before DNA extraction) might be an alternative way to specialize on various host-plants, thereby limiting the selection for adaptive loci within the genome of the insect itself. Obligate endosymbionts are widespread in insects and might play a key role in the adaptation of phytophagous insects to alternative host-plants [31].

## Conclusion

Our study shows that correlative methods are very promising to investigate the respective role of many environmental variables in shaping the geographical distribution at a locus level (allele frequency). They are particularly useful to validate outliers detected using population genetic methods, especially when populations are not at their demographic equilibrium. Finally, correlative methods represent the only way to investigate for adaptive loci when sampling is conducted at the individual rather that population level, and no *a priori *grouping of individuals into panmictic populations can be performed, rendering population genetic methods inappropriate.

## Methods

### Study sites and data collection

This study is based on a sub-sample (296 larvae) of a large AFLP dataset of great pine weevils comprising 367 individuals, including adults and larvae, collected in 20 localities throughout Europe [14,25]. Larvae were collected on either spruce (*Picea abies *Karst) or pine (*Pinus sylvestris *L. and *Pinus pinaster *Ait.) in 16 managed forest sites distributed across 4 large forestry regions: three in France (Limousin, Ardeche, and Landes) and one in Finland (Table 1). Some sites were planted only with spruce, other only with pine, and several both with spruce and pine. Three out of the four regions analysed comprised the two host-plants (Finland, Limousin, Ardeche), while the region Landes was planted only with pine (*Pinus pinaster*) (Figure 1). For each sampled site, previously geo-referenced [23], we extracted nine environmental variables (same variables used by [12]) as yearly mean of monthly values for the period 1961-2001 (available at: http://www.cru.uea.ac.uk[28]): mean diurnal temperature range (°C), number of days with ground frost, precipitation (mm/month), coefficient of variation of monthly precipitation (%), relative humidity (%), maximum possible sunshine (% of day length), mean temperature (°C), wet days (number of days with > 0.1 mm rain per month), wind speed (m/s at 10 m above the ground). The altitude of the site was also noted.

Larvae were genotyped at 83 unlinked AFLP loci as described in Conord *et al*. (2006) [25]. Fragment length ranged from 50 to 375 bp, and most fragments (86%) were longer than 100 bp limiting the probability of homoplasy (co-migrating non-homologous fragments) which is known to affect mostly small fragments [25]. AFLP patterns were then visualised with GENOGRAPHER V1.6.0 software, in which a fluorescent peak corresponds to the presence of an amplified restriction fragment. Polymorphic peaks were checked individually and a presence/absence (i.e. 1/0) matrix was constructed. A previous genetic structure analysis showed that regional forests are a pertinent geographic scale for defining populations in the large pine [25].

### Outlier detection using a population genetic approach

To detect outlier loci presenting a signature of positive selection in our AFLP dataset we used two population genetic approaches. Both methods aimed at detecting loci with a higher than expected differentiation between populations in contrasting situations (e.g. contrasting climatic conditions, and/or alternative host-plant species): (1) the popular Beaumont and Nichols [7] Dfdist program developed from Fdist for the analysis of dominant data (available at http://www.rubic.rdg.ac.uk/~mab/stuff/) and (2) a new hierarchical Bayesian method (BayeScan, http://www-leca.ujf-grenoble.fr/logiciels.htm[9] that also allows the accommodation of AFLP data. Dfidst is a frequentist method based on summary statistics of a symmetrical island model (i.e. drift-migration equilibrium, [8]. Dfdist implements the Bayesian method of Zhivotovsky [32] for dominant markers [32] to estimate allelic frequencies. Outliers were identified by plotting *F*_ST _against heterozygosity under the assumption of Hardy-Weinberg equilibrium. Significance values were obtained by generating a null distribution of *F*_ST _values based on 50 000 simulated loci with a mean *F*_ST _equal to the trimmed mean *F*_ST _calculated by removing the 30% highest and lowest *F*_ST _values observed in the empirical dataset [4,10]. Finally Dfdist plots the observed *F*_ST _value for each locus to detect those falling outside the neutral expectation given by the null distribution.

A first shortcoming of Dfdist is the possibility of detecting false positives (i.e. committing type-I errors) [33,34]. This risk, largely due to multiple tests (one at each locus), can be reduced by using a conservative significance level [10,35], as done here by adopting the smallest type I error of our analysis which is the one calculated for logistic regressions α = 0.0006 (see later the justification). It is also possible to estimate the number of false positives among detected outlier loci (= false discovery rate) for a given level of significance by applying Storey and Tibshirani's [11]*q*-value method. False discovery rate is a statistical method used in multiple hypotheses testing to correct for multiple comparisons. In our case, the method estimates the false discovery rate (or *q*-value) from the set of each locus *p*-values provided by Dfdist. For a significance level of 0.0006, the corresponding *q*-value is the expected proportion of false positives among detected outliers. We used the *q*-value package in R [36] for computations [11].

A second shortcoming of Dfdist is that it assumes that populations are at drift-migration equilibrium, which is unrealistic in most natural situations. In that context, the main advantage of BayeScan is that it estimates population-specific *F*_ST _coefficients, therefore allowing for different demographic histories and different amounts of genetic drift between populations. The method is based on a logistic regression model in which each logit value of genetic differentiation *F*_ST _(*i, j*) for locus *i *in population *j *is decomposed as a linear combination of the coefficients of the logistic regression, α*i *and β*j*, corresponding, respectively, to a locus effect and to a population effect. To identify adaptive loci, we focused on the posterior distribution of α*i*: a positive value suggests that locus *i *is subject to adaptive selection, whereas a negative value suggests that balancing selection is tending to homogenize allele frequencies over the populations. The posterior probability of locus *i *being under selection is estimated by defining two alternative models, one that includes α*i *and another that excludes it. The respective posterior probabilities of these two models are estimated using a Reversible Jump Markov Chain Monte Carlo (MCMC) approach [9]. The posterior probability that a locus is subject to selection, corresponding to *P *(α*i *≠ 0), is then estimated from the output of the MCMC by counting the number of times α*i *is included in the model. Another advantage of the Bayesian method compared with the frequentist method (Dfdist), is that it explicitly takes all loci into account in the analyses. Finally, the Bayesian approach also deals with the problem of multiple testing of a large number of genomic locations, as the number of tested loci is taken into account through the prior distribution. In this study, for the population and locus effects Gaussian priors with means of -2 and zero were used with standard deviations of 1.8 and 1, respectively, according to Beaumont and Balding [10]. The estimation of model parameters was automatically tuned on the basis of short pilot runs (10 pilot runs, length 2000). Preliminary tests indicated that a burn-in of 10 000 iterations was enough to have the MCMC converging. The sample size was set to 10 000 and the thinning interval to 50 as suggested by Foll and Gaggiotti [9], resulting in a total chain length of 500 000 iterations. Four independent runs were performed for each of the two datasets to account for the consistency of the detected outliers. The loci were ranked according to their estimated posterior probability and all loci with a value over 0.79 were retained as outliers. This corresponds to a Bayes Factor >3 as defined by Jeffreys [37], which provides substantial support for acceptation of the model.

In practice, applying population genetic approaches requires to define populations. We defined 5 different population-based structures in order to disentangle abiotic and biotic factors (Figure 1). Separate analyses were done for these 5 structures using both Dfdist and BayeScan. We firstly grouped individuals according to their geographic origin only (4 populations: Finland, Limousin, Ardeche, and Landes; population size 83, 115, 78 and 20 respectively) (structure 1, Figure 1), and secondly, according to their geographic origin and host-plant (7 populations: Finland Pine, Finland Spruce, Limousin Pine, Limousin Spruce, Ardeche Pine, Ardeche Spruce and Landes Pine; population size 38, 45, 30, 85, 28, 50 and 20 respectively) (structure 2, Figure 1). Finally, within each of the three regions where both alternative host-plants are found (Finland, Limousin, and Ardeche), pairwise comparisons of host-plant samples were performed (structure 3, 4, 5, Figure 1). Multiple independent pairwise comparisons of populations submitted to the same ecological constraints in different geographic areas allow confirmation of the involvement of a putative outlier in adaptation to contrasting situations, in our case alternative host-plants.

### Outlier detection using a correlative approach

Our objective was to assess the effect of the host-plant variable versus all the other measurable variables potentially involved in genetic differentiation. These include elevation, 9 climatic variables and geographic coordinates of the sites. Preliminary analyses have shown strong correlations between climatic variables and site geographic coordinates. We therefore decided to remove geographic coordinates since it was not possible, given our sampling design, to disentangle the effect of climate from the effect of geography. This would not affect our conclusion about the potential effect of host-plant type. To avoid correlation between the explanatory variables, a principal component analysis (PCA) was applied on the correlation matrix of climatic and elevation variables. The two first axes of the PCA represent the best way to represent variation in environmental abiotic factors at the studied scale. Multiple logistic regressions with a logit link and binomial error distribution [38] were then used to explain the presence and absence of each marker by the coordinates of the two first axes of PCA (orthogonal predictors) representing environmental abiotic variables, the host-plant variable and all the three possible interactions between these three explanatory variables. Models were fitted using a maximum likelihood method [38]. We used backwards elimination to select the variables in the final models using Akaike's information criterion (AIC). Then, for each selected model, we also tested each regression coefficient with a likelihood ratio test [38]. The significance of the likelihood ratio tests were tested using a Bonferroni correction to correct for multiple testing (significance level = 0.05/83 where 83 is the number of markers = 6 × 10^-4^). Although initially all explanatory variables were potential predictors, only those variables selected by the above criteria were used in the final models. False discovery rates were also calculated using the type I error of 0.0006. All analyses were conducted using R software [36].

### Isolation by distance pattern on neutral loci

To test for isolation by distance on neutral loci, we applied a Mantel test between genetic and Euclidian geographic distances calculated between the 16 sites. Genetic distances were computed with AFLP-SURV 1.0 [26] as pairwise *F*_ST _using the Bayesian method with non-uniform prior distribution to estimate allele frequencies with dominant markers [32]. Geographic distances (in km) and Mantel tests were calculated using R software [36]. We not only tested the relation between *F*_ST_/(1-*F*_ST_) and the geographic distance (km), but also used the logarithm of the geographic distance as recommended by Rousset [39].

## Authors' contributions

SM performed the statistical analysis and drafted the manuscript. CC participated to the design and carried out the molecular analysis. LD conceived the study, performed the population genetic analyses, coordinated and drafted the manuscript. All the authors read and approved the final manuscript.
